# LDL-Based Lipid Nanoparticle Derived for Blood Plasma Accumulates Preferentially in Atherosclerotic Plaque

**DOI:** 10.3389/fbioe.2021.794676

**Published:** 2021-12-01

**Authors:** Christian A. Boada, Assaf Zinger, Scott Rohen, Jonathan O. Martinez, Michael Evangelopoulos, Roberto Molinaro, Madeleine Lu, Ramiro Alejandro Villarreal-Leal, Federica Giordano, Manuela Sushnitha, Enrica De Rosa, Jens B. Simonsen, Sergey Shevkoplyas, Francesca Taraballi, Ennio Tasciotti

**Affiliations:** ^1^ Regenerative Medicine Program, Houston Methodist Research Institute, Houston, TX, United States; ^2^ Tecnológico de Monterrey, Escuela de Ingeniería y Ciencias, México, Mexico; ^3^ Department of Engineering Medicine, Texas A&M University, Houston, TX, United States; ^4^ Laboratory for Bioinspired NanoEngineering and Translational Therapeutics, Department of Chemical Engineering, Technion−Israel Institute of Technology, Haifa, Israel; ^5^ Regenerative Medicine Program, Houston Methodist Research Institute, Houston, TX, United States; ^6^ IRCCS Ospedale San Raffaele srl, Milan, Italy; ^7^ Department of Biomedical Engineering, University of Houston, Houston, TX, United States; ^8^ Department of Orthopedics and Sports Medicine, Houston Methodist Hospital, Houston, TX, United States; ^9^ Department of Health Technology, Technical University of Denmark, Lyngby, Denmark; ^10^ San Raffaele University, Rome and IRCCS San Raffaele Hospital, Rome, Italy

**Keywords:** LDL, Apolipoprotein, Rapamycin, Liposome, Nanoparticle, Drug Delivery, Atherosclerosis

## Abstract

Apolipoprotein-based drug delivery is a promising approach to develop safe nanoparticles capable of targeted drug delivery for various diseases. In this work, we have synthesized a lipid-based nanoparticle (NPs) that we have called “Aposomes” presenting native apolipoprotein B-100 (apoB-100), the primary protein present in Low-Density Lipoproteins (LDL) on its surface. The aposomes were synthesized from LDL isolated from blood plasma using a microfluidic approach. The synthesized aposomes had a diameter of 91 ± 4 nm and a neutral surface charge of 0.7 mV ± mV. Protein analysis using western blot and flow cytometry confirmed the presence of apoB-100 on the nanoparticle’s surface. Furthermore, Aposomes retained liposomes’ drug loading capabilities, demonstrating a prolonged release curve with ∼80% cargo release at 4 hours. Considering the natural tropism of LDL towards the atherosclerotic plaques, we evaluated the biological properties of aposomes in a mouse model of advanced atherosclerosis. We observed a ∼20-fold increase in targeting of plaques when comparing aposomes to control liposomes. Additionally, aposomes presented a favorable biocompatibility profile that showed no deviation from typical values in liver toxicity markers (i.e., LDH, ALT, AST, Cholesterol). The results of this study demonstrate the possibilities of using apolipoprotein-based approaches to create nanoparticles with active targeting capabilities and could be the basis for future cardiovascular therapies.

## Introduction

Cardiovascular disease (CVD) is the single most common cause of death worldwide and a serious public health concern for all countries, accounting for nearly one in three deaths (Virani et al., 2020). In the United States, CVD affects 48% of all adults older than 20 years of age and incurs costs of more than 350 billion dollars annually (Virani et al., 2020). The high morbidity and mortality highlight this disease’s staggering human and economic cost and the need to develop more effective treatment methods.

Nanotechnology has positioned itself as a promising tool for drug delivery through the use of synthetic nanoparticles (NP) that can carry, direct, and deliver drugs to target tissues (Fang et al., 2011; Lobatto et al., 2015; Sushnitha et al., 2020). Based on the natural tropism of LDL to accumulate in atherosclerotic plaques, apolipoprotein-based NPs using LDL represent the logical step to create drug delivery platforms for CVD. LDL-based approaches for CVD offer unique targeting specificity due to LDL’s role in the cholesterol influx pathway that allows it to localize towards plaques through surface membrane interactions between apoB-100 and scavenger receptors on activated endothelium over plaques (Mietus-Snyder et al., 1997; Han et al., 1998; Kangas et al., 1999; Cominacini et al., 2000). Moreover, LDL is characterized by prolonged circulation (Pietzsch et al., 2000) and the ability to bypass rapid detection by the mononuclear phagocytic system (Steinbrecher et al., 1984). Despite these advantages, their use as a drug delivery platform in a clinical setting is hindered by their tendency for aggregation (Hammel et al., 2003) and non-scalable purification procedures (Masquelier et al., 1986).

Advances in nanoparticle (NP) synthesis now allow the incorporation of proteins derived from cells onto the surface of lipid-based particles to create biomimetic NPs using a standardized, scalable, and reproducible microfluidic-based approach (Molinaro et al., 2016, 2018). These biomimetic NPs have the advantage of the functionalization of native proteins derived from cells or other protein sources (e.g., plasma) (Parodi et al., 2013; Molinaro et al., 2016; Palomba et al., 2016) to achieve the transplantation of hundreds of critical proteins that confer active targeting properties (Molinaro et al., 2016; Martinez et al., 2018) while maintaining drug loading properties of liposomes. Additionally, liposomal NPs have a low toxicity record backed by 20 years of use in clinical practice (Barenholz, 2012) that provides a comprehensive characterization of the pharmacokinetic properties of this technology. We hypothesize that the localized delivery of small molecules using a liposome-based nanoparticle with active targeting properties can increase the efficacy and safety of drugs already available for the treatment of cardiovascular disease. Additionally, the development of LDL-based NPs can increase the list of available therapeutics by providing a method to locally deliver novel biological therapeutics (e.g., RNA, proteins, and peptides) that remain a challenge due to poor stability upon systemic injection (Molinaro et al., 2013).

Taking cues from the molecular biology of atherosclerosis and the natural function of LDL, in this manuscript, we describe a nanoparticle that incorporates LDL on its surface that we have called “Aposomes.” The methods outlined in our manuscript overcome critical barriers in synthesizing nanoparticles using native lipoproteins to create nanoparticles. Aposomes show preferential accumulation over the atherosclerotic plaque in mice models while retaining drug loading properties. This manuscript shows a proof of concept for a nanoparticle platform that can be developed into novel therapeutics for cardiovascular disease and expanded for other diseases like liver cancer.

## Materials and Methods

### FPLC Separation of LDL

The apolipoprotein B necessary for the synthesis of aposomes was purified from blood samples discarded (a week before expiration date) by the blood donor center of Houston Methodist Hospital. The protocol was approved by the internal review committee ID: ADM00014114. The samples extracted were from patients who voluntarily visited the donor center; their identity was not disclosed to researchers. Blood was collected in 450 ml packets with EDTA to avoid coagulation. To eliminate red blood cells, the sample was centrifuged for 10 min at 5,000 rpm. The red blood cells formed a plaque at the bottom of the container, and the supernatant plasma was removed, and the rest discarded. Serum samples were aliquoted into 1 ml vials, and Halt^®^ (Thermo Fischer) protease inhibitor was added before the sample was frozen at -80 C for future use.

LDL was separated on a protocol previously outlined by Innis-Whitehouse et al. (Innis-Whitehouse et al., 1998) using the fast protein liquid chromatography (FPLC) (BioRad Duoflow) 10 equipped with a Sepharose 6 10/300 column (GE Healthcare). 1ml of whole blood plasma was injected into the system. Before injection, all samples were filtered through 0.2 um PDVF syringe filters. The column was equilibrated under isocratic flow in a buffer solution made with 0.1 M of NaCl, 0.001 M NaN3, and 0.2% EDTA for 60 min under a flow rate of 0.5 ml/min. The plasma sample was run under an isocratic flow of 0.5 ml/min for 60 min, and fractions of 1 ml were collected using BioLogic Biofraction (BioRad) equipped with a detection system capable of detecting UV 280 nm and 254 nm. Plasma was divided into 1 ml aliquots, and 10 ul of Halt^®^ (Thermo Fischer) protease inhibitor was added before the sample was frozen at -80 C for future use. The fractionation of whole blood plasma using a size exclusion column gave 30 different 1 ml samples, denominated F1-F30. As a control, LDL purified using a kit for LDL/VLDL from Cell-Biolabs inc. was used.

### Synthesis and Physicochemical Characterization of Aposomes

Aposomes were assembled using both thin-layer evaporation/extrusion (TLE) method and a microfluidic method (Precision Nanosystems) to determine the method that produced the most favorable characteristics in terms of size and surface protein functionalization of the NP formulation. The TLE was performed as previously reported by Molinaro et al. (Molinaro et al., 2016). Briefly, an aqueous solution containing apoB-100 associated with LDL (fraction 12 or 13 from the FPLC run) was used to hydrate the lipid film containing 1,2-dipalmitoyl-sn-glycerol-3-phosphocholine (DPPC), cholesterol, and 1,2-dioleoyl-sn-glycerol-3-phosphocholine (DOPC) (4:3:3 M ratio, respectively) at a total lipid concentration of 20 mg/ml. The vesicle suspension was then extruded through 200 nm filters to obtain the final NP formulation.

For the microfluidic approach, we used the Nanoassmblr™ benchtop from Precision Nanosystems following a slightly modified protocol developed from our lab (Molinaro et al., 2018). Before aposome synthesis, the system was washed according to the manufacturer’s specifications with 3:1 water to ethanol at a total flow rate of 2 ml/min. After this, the aqueous phase and the organic phase were prepared as follows. The aqueous phase consisted of a solution 1 ml of fractionated plasma, while the organic phase was constituted of a mixture of DPPC, cholesterol, and DOPC (4:3:3 M ratio, respectively) dissolved in ethanol.

For both methods, Rapamycin-loaded aposomes were prepared by adding the hydrophobic drug to the organic phase before resuspension in TLC-method and before the microfluidic mixing at a concentration of 40 ug/ml in the other case. For Internalization studies, rhodamine-DOPE (PE lissamine rhodamine B), Avanti Lipids) was added into the lipid mixture at a 0.1 M ratio. Before mixing, the organic phase was pre-heated at 45 C. The two phases flowed through the microfluidic chip at 1 ml/min total flow rate and 2:1 water to ethanol flow rate ratio. The particles’ solution’s total volume was 1 ml; particles were then stored at 4°C, and analysis was performed no more than 24 h after synthesis.

### Nanoparticle Size and Surface Charge Measurements

The structural integrity of aposomes in different storage conditions for extended periods was evaluated by size, polydispersity index, and zeta potential measurements every 24 h for 4 days. We analyzed aposomes made from FPLC fraction 12 and 13 and compared them to liposomes. For each sample, biological replicates were measured and for each replicates, half of the volume of particles was placed into room temperature storage and the other half into 4 °C storage. All samples were measured using Malvern Zetasize Nano ZS. Size and polydispersity index (P.D.I) were measured in a low volume disposable sizing cuvette (Malvern) with five ul of particles diluted in 495 ul of water 173 backscatter measurement angle. Measurement parameters were set to three measurements consisting of 10 runs of 10 s each with a general-purpose (standard resolution) analysis model. Zeta potential was measured using 10 ul of particles +900ul of deionized water 1 ml in a clear disposable zeta cell (Malvern) with measurement parameters set at three measurements, each with 20 runs using an “auto mode” analysis model.

### Cryo-TEM Grid Preparation, Imaging, and Nanoparticle Membrane Thickness Measurements

Cryo-TEM Grid Preparation and Imaging was done as previously described in the methods by Boada et al. (Boada et al., 2020). Briefly, aposomes, liposomes, and LDL NP were placed on Quantifoil holey carbon cryo-transmission electron microscopy grids (Quantifoil Micro Tools GmbH, Jena, Germany). Grids were glow discharged using an Ernest Fullam Air Glow Discharge (Ted Pella, Inc.) for 45 s with a 50 uA current and a vacuum of ∼200 psig. Images were captured using a Direct Electron DE20 direct detection device (Direct Electron, San Diego, CA). Images were all taken with ∼30e-per square angstrom per second dose rate with an exposure rate of 1s and a total of 24 frames per exposure. The angstrom per pixel values for each magnification are 3.04 and 1.64, respectively.

### Chemical Analisis of Nanoparticles Using Fourier Transform Infrared Spectroscopy 

To characterize the chemical composition of aposomes, Fourier transform infrared (FTIR) spectroscopy was used. Samples were analyzed using a Nicolet 6700 FT-IR (Thermo Scientific) with Smart iTR attachment. 10ul of samples were placed on a diamond piece, and the samples were left until all water was evaporated. Samples were measured using a data spacing of 0.241 cm–1 with a CO2 correction, and resulting spectra were processed using OMNIC Spectra Analysis software with baseline correct and smoothing functions.

### Encapsulation Efficiency of Rapamycin in Aposomes

Rapamycin encapsulation was measured using high-pressure liquid chromatography (HPLC). The experiment was performed on a Waters e2695 equipped with a UV/Vis detector module UV/Vis 2,489. The column used was a Phenomenex Luna (5 um) C18, 250 × 4.5 mm. The separation was performed under isocratic elution with a solution made of Water/Methanol 20%: 80% (v/v). The samples ran using a flow rate of 1.23 ml/min and monitored using a detection module set at 277 nm. The sample injection volume for all the samples was 10 ul, while the sample temperature was set at 10 C. Encapsulation efficiency was defined as the percentage of rapamycin encapsulated when compared to a standard solution of free rapamycin.

### Detection of Proteins on Nanoparticle Surface Using Flow Cytometry

To probe protein presence on aposomes surface, 100 ul of a 9 mM formulation of aposomes derived from previously established protocols (Molinaro et al., 2016) were taken for analysis 700 ul of 1% Fetal Bovine Serum diluted in PBS and incubated for 60 min under agitation at 700 rpm at 4C with 1/1,000 dilution of polyclonal anti-apo-B antibody (anti-apolipoprotein B FITC, Abcam ab27637), protected from light as previously reported (Molinaro et al., 2016). To eliminate unbound antibodies, the incubated sample was dialyzed in a membrane with a 1000 kDa cutoff (Float-A-Lyzer G2, Spectrum labs G235037) in 1,000 ml of deionized water under agitation protected from light, for 90 min. The sample was measured using a BD LSR Fortessa (BD Biosciences) equipped with 405, 488, 561, and 630 nm excitation lasers and the ability for 13 color detection. FITC detection was carried out using a 525/50 bandpass filters. As a control, 100 ul of a 9 nM formulation of liposomes incubated with polyclonal anti-apoB-100 antibodies were used to establish a baseline and draw threshold gates. Ten thousand detection events were used for each condition. For immunogenicity studies, after blocking, liposomes and aposomes were incubated with 100 ul of plasma (healthy and treated) to the solution and incubate for 30 min under agitation at 700 rpm at room temperature. Afterward, liposomes and aposomes were incubated separately with IgG (Invitrogen, F-2761) or IgM (Invitrogen, A-21238 Catalog Number) Ab for 60 min under agitation at 700 rpm at room temperature.

### SDS-PAGE and Western Blot Analysis

Analyzed samples were dissolved with 1x Laemmli Sample Buffer (Bio-Rad Laboratories, Hercules, CA United States) containing 2-mercaptoethanol (Sigma-Aldrich) and heated for 5 min. At 95°C. These samples were loaded onto 4–15% Mini-PROTEAN^®^ TGX™ Precast Protein Gels (Bio-Rad) and run for 2 h at 100 V. For whole protein detection. The gel was stained with SimplyBlue™ SafeStain (Thermo Fisher Scientific) overnight and wash with distilled water. For each apoB-100 and apoA-I detection, proteins on the gel were transferred to the *Trans*-Blot^®^ Turbo™ Mini Nitrocellulose membrane (Bio-Rad). Western blot was performed by using mouse anti-human apoB-100 primary monoclonal antibody (MAB4124, R&D Systems, 1:333 dilution) and rabbit anti-human apoA-I primary monoclonal antibody (MAB36641, R&D Systems, 1:2,000 dilution). Their corresponding IgG conjugated with horseradish peroxidase (HRP) was used for secondary detection. The membrane was developed with Clarity™ Western ECL Substrate (Bio-Rad). Protein bands were detected by ChemiDoc™ XRS + System (Bio-Rad). Each western blot result was obtained by merging with the picture of a molecular weight ladder.

### Preparation and Staining of Histology Samples From Mice Tissue

Organs were collected and sliced longitudinally with a size 20 blade (Aspen Surgical Products, Inc.). One half of each organ was placed in a tissue cassette (Fisherbrand^©^ Sure Tek-2), while the other half was returned to a 50 ml polypropylene tube containing 10% neutral buffered formalin. Each cassette contained the organs of one mouse, respectively, for all cassettes. Cassettes were then placed in sample containers containing 10% neutral buffered formalin and sent to be processed by the Comparative Medicine Pathology department at Houston Methodist Research Institute for H&E staining. Briefly, Hematoxylin and Eosin staining were performed using the ST Infinity H&E Staining System (Leica Biosystems) in Leica Autostainer ST5010 XL. Paraffin was melted before staining by heating the slides at 60°C for 30 min, then deparaffinized by performing 3 x 2-min washes in xylene, 3 x 1-min washes in 100% ethanol, 1 x 1-min washes in 95% ethanol before rinsing in tap water. Slides were incubated for 30 s in Hemalast, for 5 min in Hematoxylin, and were rinsed for 1 min in tap water. Next, slides were incubated for 30 s in Differentiator and 1 min in Bluing agent, with each step followed by a tap water rinse for 1 min. Then 95% ethanol for 1 min. Slides were stained with Eosin for 30 s, dehydrated in 95% ethanol for 1 × 1 minute, 1 × 4 minute in 100% ethanol, 2 × 1 min in 100% ethanol, and cleared in 3 × 2 min in xylene. Every step after the initial heating of the slides is done at room temperature. Once stained, slides were scanned using EVOS imaging software (Life Technologies) with ×20 objective magnification.

### Semi-Automated Morphological Analysis of Tissue Samples

Using FIJI (Fiji Is Just ImageJ)(Schindelin et al., 2012) image processing software, images from the lungs, heart, and liver were analyzed using custom macros (Supplementary information #2). Briefly, the cell counting algorithm consisted of an image threshold to increase the contrast between nuclei and surrounding tissue. After this step, the image was cropped to exclude tissue that was not parenchyma that might introduce artifacts in the cell count, and only tissue parenchyma was analyzed for lung, heart, and liver. After tissue parenchyma was selected, the image was thresholded to separate nuclei from other surrounding structures, and then nuclei were counted using the “Analyze Particles” function in FIJI. Values for cellularity and interstitial thickness were obtained by averaging the values from at least three different images from each mouse, and for each condition at least, three different mice were analyzed. Individual points on the violin plots represent the average for individual mice. Statistical significance was determined using a one-way ANOVA unmatched analysis with a single pooled variance and Tukey pairwise comparisons among groups within individual data sets.

### Preparation of RBC and Nanoparticle Suspensions and Measurements of Artificial Microvascular Network Perfusion Rate

Three units of packed RBCs were purchased from a local blood bank (Gulf Coast Blood Bank, Houston, TX) and kept at 4 °C until use. RBCs were adjusted to 48% hematocrit with sterile PBS before adding NP solution at 100 µL NPS to 500 µL RBCs for a final HCT of approximately 40% (39.92 ± 0.25). RBC and NP suspensions were then allowed to incubate at 37°C for 1 hour in a bead bath (Bead Bath; Lab Armor, Cornelius, OR) before being run on the AMVN. HCT was measured using a hematology analyzer (XS-1000i, Sysmex America, Lincolnshire, IL).

The design and fabrication of the artificial microvascular network (AMVN) device have been previously described in detail (Burns et al., 2012; Sosa et al., 2014). To perform the AMVN perfusion rate measurement, 35 µL of HCT adjusted RBC with NP suspension was pipetted into each of the three inlets of the device, and the device was placed on an inverted bright-field microscope (IX81, Olympus America, Inc., Center Valley, PA) equipped with a high-speed camera (Flea3, Point Grey Research, Inc., Richmond, Canada). A small pressure difference was applied to initially fill the AMVN device with the RBC suspensions. The pressure difference was zeroed to stop the flow, the field of view of the microscope was aligned with the postcapillary venules (outlet microchannels) of the networks, and image acquisition was initiated (in a burst of 10 frames at 100 fps every 10 s). After approximately 50 s, a driving pressure difference of 20 cmH2O was applied and kept constant for the duration of the experiment (3 min total). A custom program (MATLAB; The Math Works, Inc., Natick, MA) was used to find the average cell velocity in each channel. Liposome samples were pipetted into the middle inlet, while aposome samples were pipetted into the outer two networks (for a total of three independent measurements per liposome and six independent measurements per aposome per unit). Statistical significance (*p* < 0.05) in paired data between the measurements of the samples from different RBC units was calculated using a two-tailed paired Student’s t-test. Values are presented as means ± standard deviation.

#### Measurements of Elongation Index

Cell deformability was measured using a conventional LORRCA ektacytometry following the manufacturer’s specifications (RR Mechatronics, Hoorn, Netherlands)(Levi, 2004). Briefly, 25 µL of 40% HCT blood sample was pipetted into a proprietary buffered viscous polyvinylpyrrolidone solution and inverted until homogenously mixed. The cells underwent increasing shear stress from 0 to 30 Pa, while the elongation index was measured at constant temperature (37°C). Statistical significance (*p* < 0.05) in paired data between the measurement of the samples from different RBC units was calculated using a two-tailed paired Student’s t-test. Values are presented as means ± standard deviation.

### MTT Assay to Determine Nanoparticle Toxicity

Human Umbilical Vein Endothelial Cells (huvec), Murine Macrophages (J774), and Human Macrophages (THP-1) were grown in 96-well cell culture plates until 80% confluent, exposed to liposomes and aposomes for 24 h at different lipid molar concentrations of 200 nM, 400 nM, 800 nM, and 1.5 mM representing IC25, IC50, and IC100, and *in-vivo* treatment, then washed with PBS. Appropriate growth media was used as a negative control, while 1% Triton-X in PBS was used as a positive control. Cell viability was determined by adding MTT in fresh medium at a 500 μg/ml final concentration. After incubation for 2 h at 37°C, the supernatant was removed, and formazan crystals were resuspended by introducing PBS for 30 min. The absorbance was measured at 570 nm in a multiwell plate reader.

### Serum Metabolic Panel

Whole blood samples obtained from apo E −/− mice were centrifuged at 10,000 x g for 10 min at 4°C. After separation, the serum layer was carefully collected and used for further analysis. Commercially available biochemical assays were used to analyze serum concentrations of glucose (Abcam, ab65333), lactate dehydrogenase (Abcam, ab102526), alanine aminotransferase (Abcam, MAK052-1 KT), aspartate aminotransferase (Abcam, MAK055-1 KT), and cholesterol (Abcam, ab65390). 2–3 µl of serum was used for each analysis and quantified following manufacturer protocols. Glucose, lactate dehydrogenase, alanine aminotransferase groups were analyzed independently using One-Way ANOVA (sample distribution was normal for all groups) unmatched analysis with a single pooled variance for each data set, and a Tukey correction for pairwise comparisons only within the groups for individual data sets. The only exception was LDH levels, which were analyzed using an unpaired Kruskal-Wallis test with Dunn’s multiple comparisons test since the sample did not present a normal distribution.

### *In-vivo* Long-Term Immunogenicity of Aposome Nanoparticles

To analyze the safety profile of aposomes and immune response *in-vivo*, healthy mice were injected retro-orbital with 200ul NPs once a week for 4 weeks for a total dose of 1000 mgkg^−1^. Mice were sacked after 8 weeks, and their blood was collected to observe any humoral response. The collected blood was placed in an Eppendorf tube containing 10% total volume of heparin. The tubes containing blood were spun down at 3,000 rpm for 15 min in a centrifuge. After centrifugation, the supernatant was collected and placed in a separate tube; the remaining red blood cells were discarded. To the samples, Halt protease inhibitor was added (Thermo Fischer 87,785) and then stored in 2C freezers to be analyzed no later than 3 days after the experiments. The protocol was reviewed and approved by Methodist hospital internal review board (ID: Pro00012800).

### *In-vivo* Analysis of Biodistribution and Plaque Targeting

For plaque targeting experiments, apo e −/− mice that spontaneously develop plaques after 12 weeks on a high-fat diet were used. After 12 weeks, mice (n = 3) were injected retroorbitally with 166 µL of a 9 mM solution of rhodamine-labeled liposomes or aposomes. Some mice were injected with anti CD68 30 min previous to NP injections (Abcam, ab27637).

After 2 h, mice were sacrificed, and their aorta was removed from the base of the heart to its bifurcation into the iliac arteries, cut longitudinally, and placed on a Petri dish with the plaques exposed. Plaques were imaged using upright Nikon A1R laser scanning confocal microscope with a resonance scanner, motorized and heated stage, and Nikon long-working distance ×4 and ×20 dry objectives. The microscope is housed within the Intravital Microscopy Core at HMRI. At least three vascular lesions were analyzed per mice for both liposomes and aposome groups. Images were quantified using Nikon Elements, and the accumulation of particles was directly correlated to the intensity of the rhodamine (i.e., red) signal in the resulting images. Images were quantified using FIJI (ImageJ) software available from NIH. The accumulation of particles was directly correlated to the intensity of the rhodamine (i.e., red) signal in the resulting images w/background fluorescence removed for all groups. The plaque area was calculated by drawing an ROI around the vascular lesion and measuring its surface area. The entire data set was analyzed using ordinary one-way ANOVA with Tukey’s multiple comparison test. All statistical analysis was performed using Graphpad Prism software.

ApoE (−/−) mice fed with a high-fat diet for 12 weeks were used for these biodistribution studies. After these 12 weeks, mice were injected with 166 uL of 9 mM rhodamine-labeled liposomes or aposomes. Two hours following the injection of NPs, mice were sacrificed, and organs were collected. *Ex vivo* imaging of organs (Heart, lung, liver, spleen, kidneys, and aorta) and injected particles was performed using an IVIS^®^ spectrum. Image acquisition parameters included the following: excitation = 535 nm, emission = 600 nm, exposure time = 0.5 s, binning = 4 and f/stop = 2. Images were exported and analyzed using Living Image^®^ software. ROIs were drawn around individual organs, and radiant efficiency of the signal was quantified. % injected dose was calculated as [(radiant efficiency in an organ)/(radiant efficiency of particles)]*100.

## Results

### Microfluidic Methods Are Better Suited for Protein Incorporation Into Nanoparticle Surface Than Conventional Thin-Layer Evaporation

**FIGURE 1:**
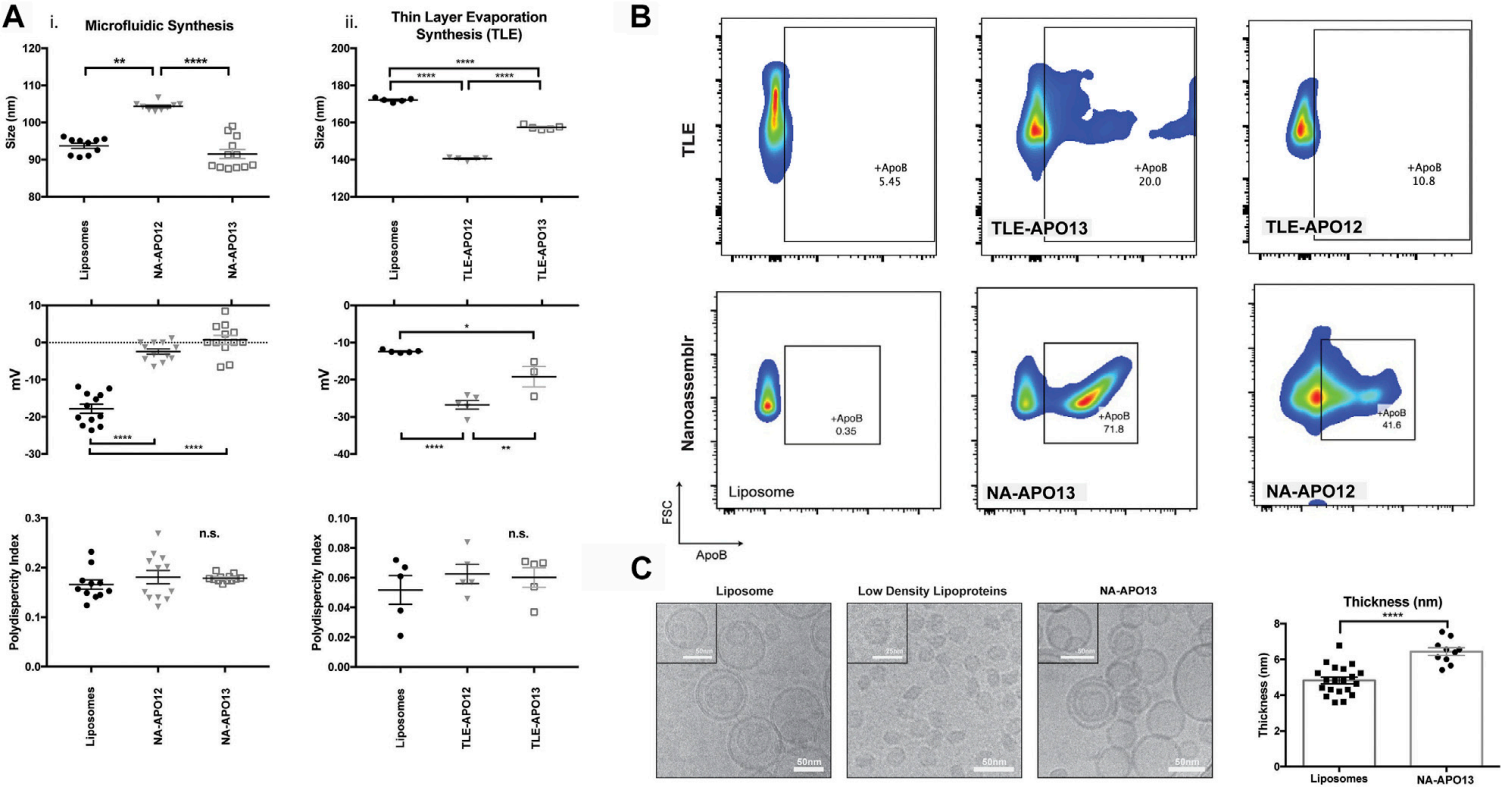
Aposome nanoparticles made using nanofluidic synthesis method show higher apoB-100 enrichment of nanoparticles compared to TLE method – **(A)** Size, polydispersity index and surface charge for nanoparticles (NPs) made using a nanofluidic method and thin layer evaporation using two different protein sources for LDL derived from two different plasma fractions **(B)** Flow cytometry analysis of surface apoB-100 show that NPs made using a microfluidic method show higher LDL enrichment than similar particles synthesized using thin layer evaporation, based on these results it was determined that particles made with FPLC fraction 13 and using a microfluidic method was most effective for LDL enrichment. From this point onward Aposome refers to these particles unless specified otherwise **(C)** Cryo-TEM imaging of liposomes and Aposomes, Aposomes show increased membrane thickness of the lipid bilayer assessed using Fiji software.

Methods for reconstituting membrane-bound proteins into the membrane of liposomes have typically included thin layer evaporation (TLE) in combination with extrusion (Molinaro et al., 2016; Zinger et al., 2018) and detergent depletion (Rigaud and Lévy, 2003). We tested the TLE method against microfluidic synthesis to compare the physical characteristics of the resulting NPs, such as size, polydispersity index, and surface charge, as well as apoB-100 incorporation efficiency. For both approaches, we used two different fractions derived from blood plasma using size exclusion chromatography (SEC) selected for their high cholesterol content, which correlates with LDL concentration (Innis-Whitehouse et al., 1998). Using TLE, the resulting nanoparticles using F12 as a source of apoB-100 through TLE and extrusion method (TLE-APO12) yielded particles with a size of 140 nm+/−1 nm PDI of 0.1±0.02 and a surface charge of −26.8 mV+/−2.6 (Figure 1A.). For aposomes synthesized using fraction 13 (TLE-APO13), size was 157 nm+/−1 nm with a PDI of 0.06±0.02 and a surface charge of -19 mV+/−5 mV. In contrast to TLE-manufactured NPs, aposomes synthesized using a microfluidic method, namely a NanoAssemblr® (NA), were significantly smaller with a size of 90 nm+/−8.5 for liposomes and 100 nm+/−7 nm for F12 aposomes (NA-APO12) and 91 nm+/−4 nm for F13 aposomes (NA-APO13) (Figure 1A.). PDI for particles synthesized using the microfluidic methods were slightly more polydisperse, but well below the acceptable standard of 0.2 (NA-APO12 - 0.18, NA-APO13 - 0.17). Surface charge measurements of nanoparticles varied when using different synthesis methods. NPs made with microfludic methods had a higher charge, NA-APO13 presented an average surface charge of −2.4 mV+/−3 mV and NA-APO13 presented an average charge of 0.7 mV+/−4 mV compared to −27 mV+/−3 mV for TLE-APO12.

To assess apoB-100 enrichment on the surface of aposomes, we incubated NPs with an anti-apoB-100 FITC (fluorophore)-labeled antibody and measured the corresponding fluorescence through flow cytometry using liposomes as a negative control. Results from flow cytometry demonstrated that TLE NPs were only capable of 10.8% enrichment using fraction 12 and 20% enrichment using fraction 13. In contrast, microfluidic synthesis was much more efficient at incorporating apoB-100 on the surface of aposomes with fraction 13 (NA-APO13), enriching the highest percentage of 71.8% (40% for NA-APO12 - Figure 1B). Based on these results, we concluded that the most efficient method to incorporate apoB-100 onto the surface of lipid-based particles was using a microfluidic synthesis method using fraction 13 of SEC purified plasma. Therefore, from here onwards, all experiments were performed using NA-Apo13 and will be referred to as aposome unless otherwise noted.

**FIGURE 2:**
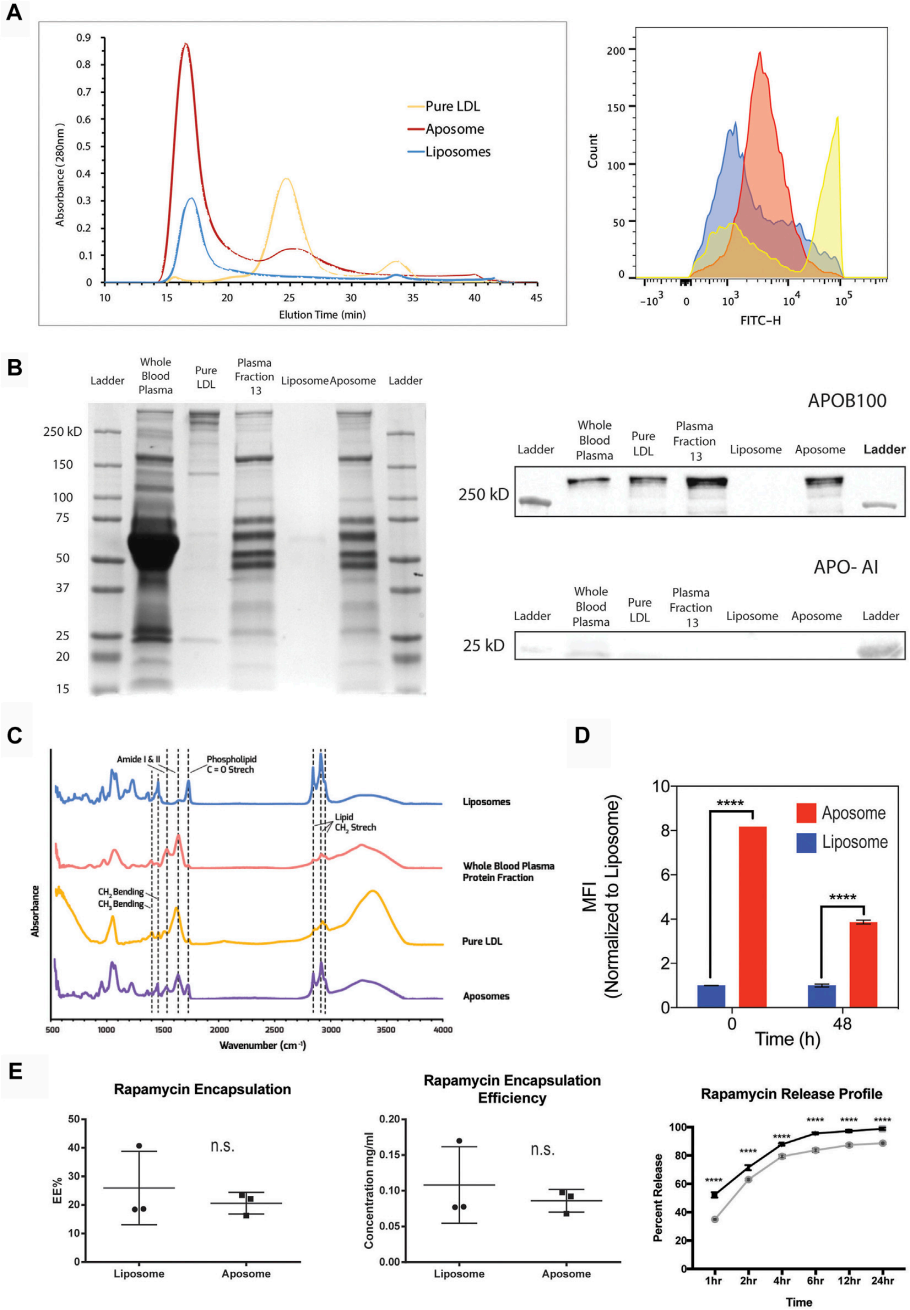
**-** Aposome nanoparticles show higher apo-B100 enrichment of nanoparticles and prolonged drug release capability **(A)** FPLC profile of aposomes shows retention of apoB-100 after size exclusion chromatography; increased absorbance is indicative of increased protein presence. Corresponding flow cytometry (G) of chromatographic peaks confirm retention of apoB-100 **(B)** SDS-PAGE of aposome NP maintains protein bands from plasma fractions, especially those above 250 kDa, indicative of apoB-100 presence. Western blot of aposome particles confirms apoB-100 presence in aposomes samples **(C)** FTIR of aposomes shows peaks that are consistent with both liposomes and pure LDL, corroborating, as well, the presence of apo-B100 in aposomes **(D)** Flow cytometry of long-term retention of apoB-100 aposomes shows that after 48 h aposomes retain LDL **(E)** Aposomes is capable to encapsulate a small hydrophobic molecule (i.e., Rapamycin) with a prolonged release curve as was assessed by HPLC.

Cryo-EM was performed on aposomes and compared to liposomes and LDL to analyze aposome morphology. Qualitative analysis of images demonstrated that aposomes had a spherical structure consistent with lipid-based NP with a similar bilayer membrane structure. Interestingly, aposomes showed significantly increased membrane thickness (6±1 nm) when compared to liposomes (5±1 nm) (Figure 1C). Increased thickness was likely due to the incorporation of apoB-100 within the lipid bilayer of aposomes, in line with our previous observations (Molinaro et al., 2016). Fourier transform infrared spectroscopy (FTIR) was performed to corroborate the presence of apoB-100 on the surface of these particles. FTIR showed that aposomes haves spectrum peaks typical of lipids signal at 2,700 and 2,800 cm^−1^, characteristic of CH_3_ stretch with an additional peak at 1800 cm^−1^ that corresponds to phospholipid C=O stretch (as for liposomes). Lastly, the presence of peaks at 1,500 to 1700 cm^−1^, where amine groups signal would be expected, was absent in liposome profiles but present in that of LDL (Figure 2C), providing further indirect proof of protein presence on aposomes (Petibois et al., 2001).

In addition to the morphological analysis, we measured the ability of aposomes to retain apoB-100 on their surface. We used SEC combined with fast protein liquid chromatography (FPLC) to test protein retention and verified results of protein enrichment using flow cytometry. FPLC chromatography profiles show that aposomes present a significant peak at 17 min like liposomes. This peak corroborates DLS results as liposomes and aposomes are both larger than ∼30 nm (the limit size separation for a Sepharose six column) as they elute at the void fraction. However, aposomes show a higher absorbance than liposomes, likely a result of protein incorporation on the surface of aposomes. A lower secondary peak seen in the aposomes’ chromatography profile appears at 25 min, like the peak observed in LDL samples; this likely corresponds to a small fraction of LDL that was not incorporated into aposomes. (Figure 2A).

Given that the initial particle/protein concentration was matched, the initial peak in the aposome’s chromatograph corresponds to aposomes with apoB-100 incorporated in their bilayer. In contrast, the second peak is likely free, unincorporated apoB-100/LDL. To corroborate this, flow cytometry with antibodies against apoB-100 was performed on fractions obtained from the peaks of the FPLC profile. Results show that the chromatographs corroborate the results seen from FPLC with aposomes showing enrichment after FPLC fractionation (Figure 2A). These results strengthen the argument that aposomes incorporated apoB-100 within their lipid bilayer and retained this protein after being subjected to pressure and shear stress of the FPLC. Furthermore, the results strongly suggest that aposomes have LDL embedded in their bilayer and not just an aggregation of LDL and liposomes.

Aposomes were further characterized using SDS-PAGE and western blot to profile the presence of LDL in nanoparticle samples qualitatively. SDS-PAGE was performed on whole blood plasma, plasma fraction 13, and aposomes; liposomes were used as a negative control, while LDL was used as a positive control for apoB-100. Results show that aposomes can preserve all of the significant protein bands seen in the SDS-PAGE, with bands above 250 kDa, characteristic of apoB-100 present in whole blood plasma, fraction 13 plasma, and aposomes. In addition, apoA-I predominant apolipoprotein in molecules such as HDL was strongly detected in only whole blood plasma, suggesting that gel chromatography is effective in separating different classes of apolipoprotein on size alone (Figure 2B).

To evaluate the drug delivery potential of the aposomes, it was critical to assess the pharmacokinetics using a model therapeutic compound. Therefore, we analyzed the drug loading capabilities of aposomes to encapsulate rapamycin, a hydrophobic compound that can reduce macrophage proliferation in atherosclerosis. Loading efficiency was measured with the total amount loaded and the release curve through 24 h using high-pressure liquid chromatography (HPLC). Results from these studies reveal that aposomes were capable of encapsulating rapamycin with an efficiency of 20% +/− 4% (Figure 2E). Altogether, aposomes show no significant difference in loading efficiency compared to liposomes, thus indicating that the incorporation of apoB-100 does not affect drug loading capabilities and aposomes retain pharmacodynamics like that of liposomes.

Additionally, the drug release kinetics were measured for aposomes showing 83% +/− 0.8% release at 4 h s. Overall, the release curve of rapamycin for aposomes and liposomes was significantly different, with aposomes releasing their cargo slightly faster than liposomes (Figure 2E). The increase in release rate is most likely due to steric competition of rapamycin and the apoB-100 protein within the lipid bilayer of aposomes. Because of its hydrophobicity, rapamycin is loaded within the hydrophobic tails of phospholipids in competition for space with apoB-100.

### Aposomes Present a Favorable Toxicity Profile *in vivo* and *in vitro*

After verifying the physicochemical properties of aposomes, we tested their safety profile *in vitro* on red blood cells (RBCs). We performed RBC deformability as a marker for testing toxicity of aposomes since they are directly injected into the bloodstream, and any alteration in RBC shape could affect gas exchange. To validate the safety of this interaction, we perfused RBCs incubated with either aposomes or liposomes through the artificial microvascular network (AMVN) device and measured the bulk perfusion flow rate. We observed that the perfusion rate for liposome-treated RBCs was 0.181 ± 0.014 nL/s, and for aposome-treated RBCs was 0.177 ± 0.009 nL/s. There was no statistically significant difference between the samples (paired *t*-test two-tailed *p* = 0.240), indicating no measurable difference for aposomes, indicating no measurable adverse effect (Figure 4B). Additionally, we assessed RBC deformability by measuring the ability of RBCs to elongate in response to shear in a highly viscous environment via conventional ektacytometry (LORRCA, RR Mechatronics, Hoorn, Netherlands)(Sosa et al., 2014). The Elongation Index (EI) measured for a wide range of shear stresses (0.3–30 Pa) showed no significant differences between liposome-treated and aposome-treated RBCs (*p* > 0.05 at all shear stresses, Figure 4C). Together, these findings provide strong evidence that aposomes do not negatively affect RBC deformability. The benign nature of aposomes is indicated by both the ektacytometry assay and by the ability of RBCs to perfuse through an artificial microvascular network normally.

Toxicity was tested in additional cell types; human umbilical vascular endothelial (huvec) cells and J774 macrophages using an *in vitro* MTT assay. These 2 cell lines were chosen to further validate aposomes biocompatibility on human cells (huvec) and specifically because endothelial cells and macrophages are the main cell types that aposomes will interact with upon injection. Aposomes showed no signs of decreased viability in both cell lines compared to liposomes, even at high concentrations (Figure 4D).

### Aposomes Show a Higher Accumulation in Atherosclerotic Plaques When Compared to Liposomes *in vivo*

The potential of aposomes *in vivo* to localize over atherosclerotic plaques was investigated in a mouse model of advanced atherosclerotic plaque to show that aposomes could efficiently target plaques. ApoB-100 has a vital role in the progression of atherosclerosis, and, specifically, LDL is known to accumulate in atherosclerotic plaque through scavenger receptors (Moore, 2006; Mehta et al., 2007).

We first investigated the biodistribution profile of aposomes when systemically injected in ApoE^−/−^ mice, a well-established model for late-stage atherosclerosis. Notably, we evaluated aposomes and liposome accumulation in major filtering organs (e.g., liver, spleen, lungs, and kidneys), heart, and aorta 2 hours after intravenous administration. In the organs analyzed, a macroscopic analysis using near-infrared imaging of organs did not show a significant difference in biodistribution between aposomes and liposomes (Supplementary Figure S1). For both aposomes and liposomes, most particles localize towards the liver and kidneys, despite aposomes overall accumulating slightly less in the liver and spleen than liposomes.

**FIGURE 3:**
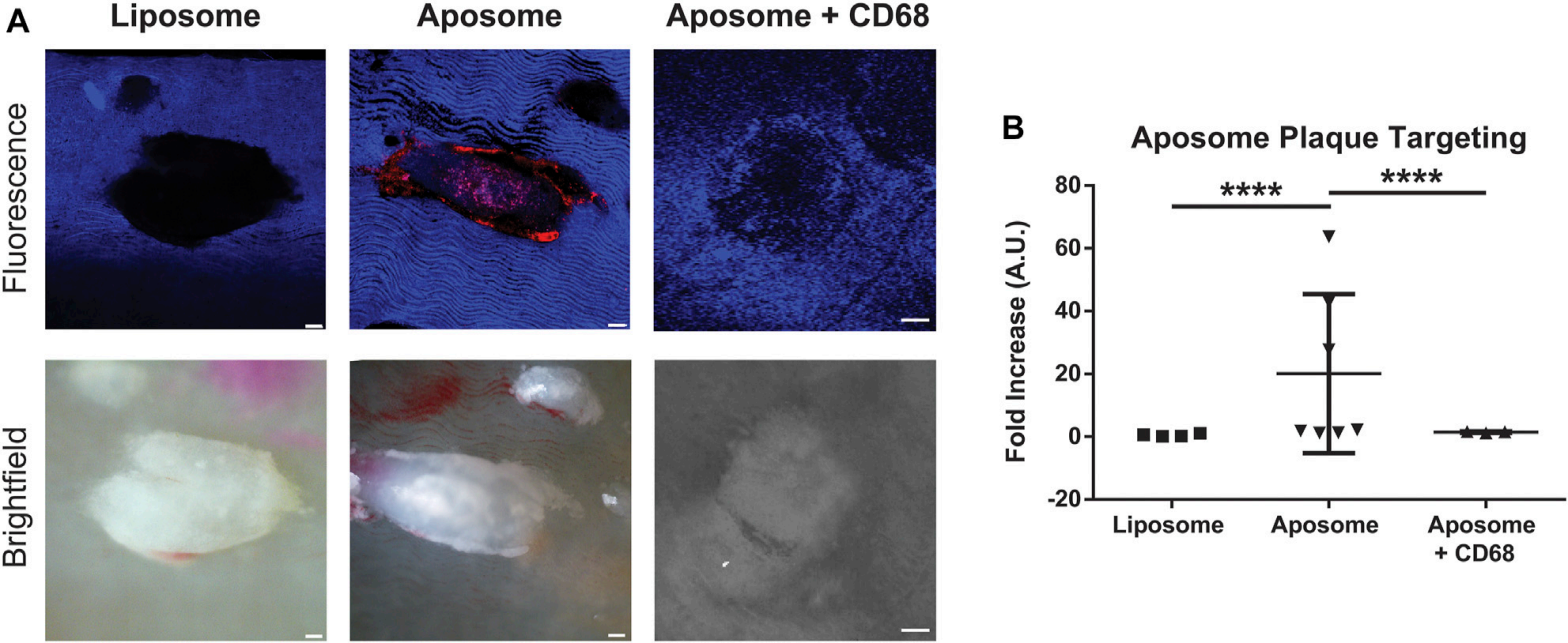
Aposomes accumulate in atherosclerotic plaques on average 20-fold more than liposomes via CD68-dependent mechanism **(A)** Confocal and bright-field images of en-face preparation of dissected aortas showing liposome and aposome plaque targeting. Liposome and aposome particles were labeled with rhodamine, which shows up as red color in the confocal image **(B)** Quantification of plaque targeting of aposomes and liposomes shows that aposomes accumulate, on average, about 20-fold more in plaques that aposomes. Injection of CD68 antibody previous to aposome injection significantly decreased aposome accumulation in plaque, suggesting that accumulation over plaque is primarily dependent on the CD68-mediated mechanism. Scale bar = 300 um.

We investigated the targeted accumulation of aposomes at atherosclerotic plaques of apoE^−/-^ mice 2 h post-injection using *ex vivo* confocal microscopy. It was hypothesized that the accumulation profile over the atherosclerotic plaque through scavenger receptors (Lu et al., 2009). The *en-face* visualization of aortas using confocal microscopy showed that aposomes had significantly increased accumulation within plaques compared to liposomes (Figure 3B). Aposomes exhibited a 19-fold increase in plaque accumulation (20.09±19.7) compared to liposomes (1.17±2.35) (*p* < 0.0001).

To understand the role of macrophages in the accumulation of aposomes on the surface of atherosclerotic plaques anti-CD68 antibody, a well-known macrophage marker conjugated with a fluorophore, was injected to understand whether aposomes accumulate directly in these cells. Interestingly, when aposomes were injected with anti-CD68 antibody, there was a significant decrease in aposome accumulation in plaques (1.47±1.21), suggesting that this antibody blocked CD-68 dependent aposome accumulation at the atherosclerotic plaque.

### Aposomes Show Low Immunogenicity With Low IgG and IgM Titers After Repeated Exposure and do Not Alter Liver Serum Markers or Tissue Morphology

**FIGURE 4:**
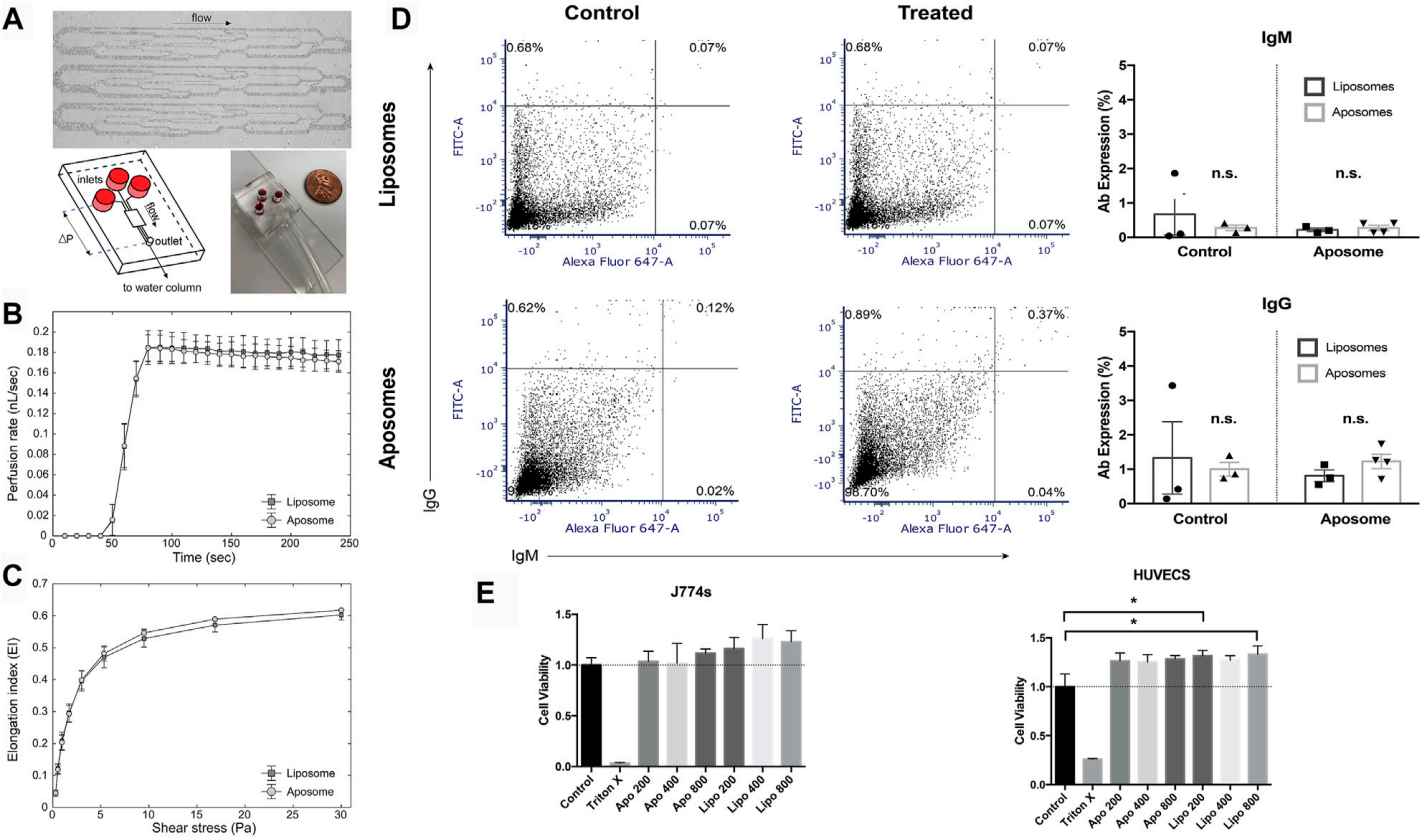
Aposomes show a favorable biocompatibility **(A)** The AMVN microfluidic device consists of thirty-four 5 µm deep microchannels ranging in width from 5 to 70 µm (to mimic arteriolar to capillary vessel size). Each AMVN has three networks connected to its independent inlet and a single common outlet that allows for the measurement of three separate samples in parallel **(B)** The AMVN perfusion rate was measured for RBCs incubated with liposome (control; n = 3) and aposome (n = 3). For each AMVN device, one liposome and two aposome samples were measured, allowing for three liposome-treated sample measurements and six aposome-treated measurements per RBC unit and nine liposome-treated measurements and eighteen aposome-treated measurements in total. The plateau regions of perfusion rates were averaged to yield the values of the mean perfusion rate (liposome: 0.18 ± 0.01 nL/s; aposome: 0.18 ± 0.01 nL/s). The differences between both groups’ mean AMVN perfusion rates were not statistically significant (paired *t*-test two-tailed; *p* > 0.05) **(C)** RBC deformability was also measured using conventional ektacytometry. RBC’s treated with either liposome or aposome underwent a range of shear stresses while ektacytometry measured elongation index (EI). The EI was not significantly different between the two groups for all shear stresses applied (paired *t*-test two-tailed; *p* > 0.05) **(D)** Mice injected with aposomes show no signs of immunogenicity as measured in the formation aposome-specific of acute or chronic antibody response as measured using flow cytometry **(E)** *In-vitro* analysis of toxicity shows no decreased viability in J774 macrophages or HUVECs (human umbilical cord endothelial cells).

The *in vivo* biocompatibility and safety are crucial for any drug delivery platform. For this purpose, biocompatibility was analyzed as previously reported (Molinaro et al., 2016). Briefly, mice were injected with liposomes or aposomes at a high dose of particles per mouse once a week and a duration of 4 weeks. The total dose administered was 800 mg kg^−1^ molar weight of lipids per mouse. After this series of injections, serum was collected, and liposomes and aposomes were incubated with the serum of mice injected with liposomes or aposomes. Particles were also incubated with serum from the control; naïve mice were not injected with either particle. The autologous IgG and IgM titers on the surface of the incubated particles were measured using flow cytometry. IgM-labeled particles, which reflect the amount of low-affinity, and less-specific antibodies generated towards the NPs, were present at low levels (Figure 4D). IgM levels of aposomes were remarkably like those in liposomes, with no significant difference between groups. IgG levels were measured to assess the adaptive immunity response towards aposomes. IgG levels were not statistically different in mice injected with aposomes when compared to liposomes. IgG adhesion on aposomes incubated with plasma from treated mice presents a 1.23±0.42% positive sample, while untreated mice presented a 1.00±0.35% positive signal for IgG. Statistical comparisons between liposomes and aposomes show no statistically significant difference between autologous IgG levels in aposomes and liposomes. Additionally, the overall percentage of positive signals for IgG and IgM levels were meager even after prolonged (1 month) administration of aposomes, showing that aposomes do not develop specific acute phase or adaptive response antibodies.

**FIGURE 5:**
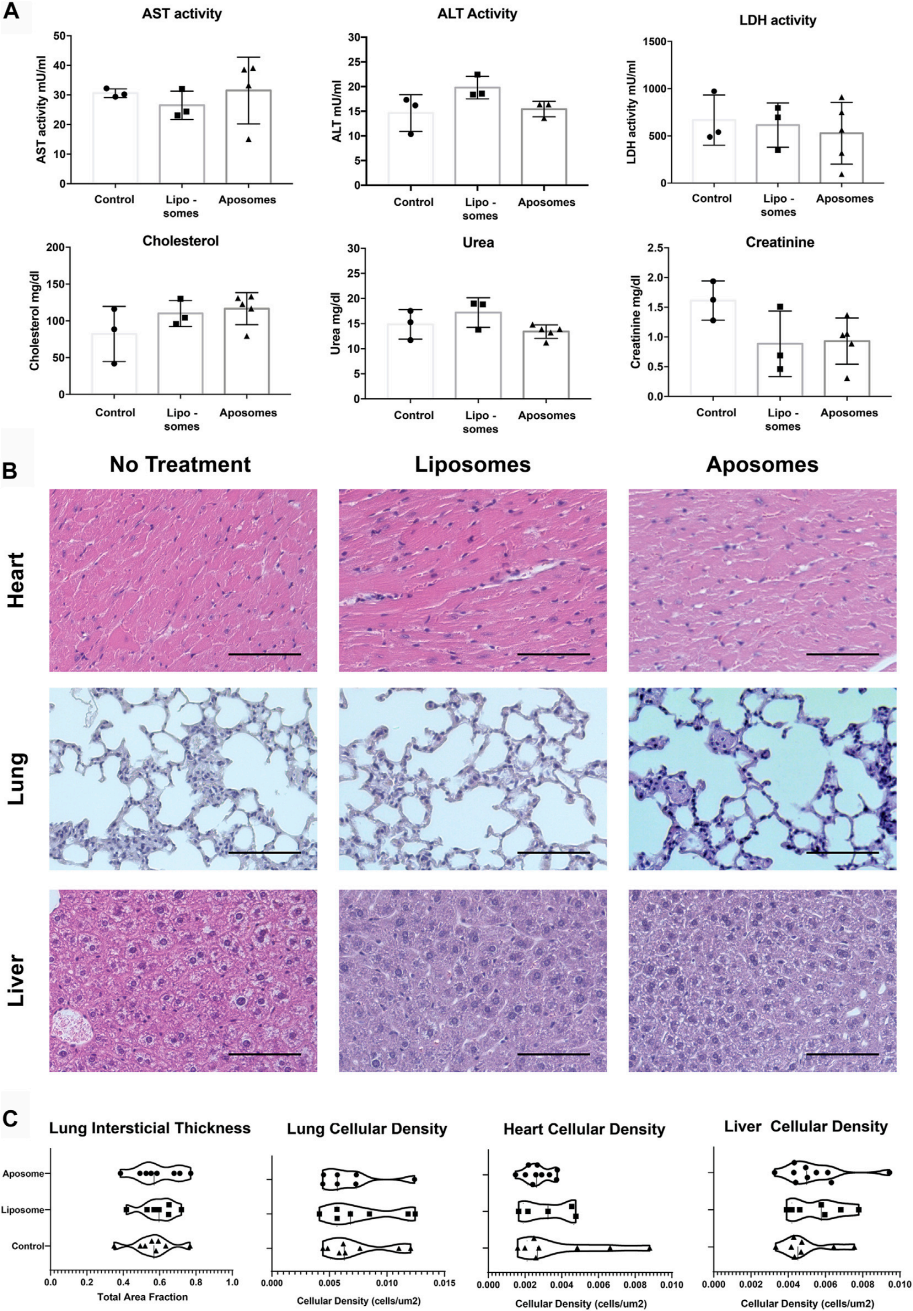
Injected aposomes induce no observable alterations of metabolic panel or tissue macrostructure in mice – **(A)** metabolic panel of mice injected with aposomes show no alterations in key metabolic markers, corroborating the biocompatibility of aposomes **(B)** H&E staining of heart, lung, liver tissue shows no abnormalities of interest **(C)** Semiautomated morphological analysis of heart, lung, and liver show no significant difference in cellular density among groups with no treatment and those treated with liposomes and aposomes. Scale bars = 100 um.

Subsequently, we analyzed the metabolic profile of mice injected with aposomes using a test panel to determine serum levels of AST, ALT, LDH, cholesterol, urea, and creatinine to analyze possible alterations in kidney and liver function. There was no statistically significant increase in the observed AST, ALT, or LDH activity in any mice treated with aposomes compared to control or liposome groups (Figure 5A). In addition, there was no significant increase in urea or creatinine for the mice treated with aposomes, signaling normal kidney function. Additionally, even though LDL is a cholesterol carrier, there was no significant increase in cholesterol levels, disproving the theory that the injection of an LDL-based nanoplatform might increase cholesterol levels, even after injection with a high dose of this platform (Figure 5A).

Subtle differences in tissue architecture are often a sensitive indicator of toxicity. Therefore, to probe tissue morphology in a detailed and objective manner, we used semi-automated quantification previously developed by our group (24,31) on H&E images. The tissue parameters were focused on the following features: cellularity within the tissue for lung, heart, and liver that could indicate non-specific cellular infiltrate. Additionally, the interstitial thickness was analyzed for lung parenchyma to determine if aposomes caused any changes that might affect oxygen exchange in the lungs. From the results, it was evident that there was no significant difference in cellularity or interstitial thickness in mice injected with aposomes compared to mice injected with liposomes or mice with no treatment (Figure 5C). Together these results show a bio-hybrid platform that showcases low immunogenicity and no alterations of metabolic profile, especially cholesterol, showing extremely favorable biocompatibility *in vivo* in mice.

## Discussion

This study represents a proof-of-concept for the functionalization of native LDL, in particular apoB-100, into a lipid-based hybrid NP using a microfluidic fabrication method. Long established protocols to purify LDL relied on ultracentrifugation-based methods that are labor-intensive, require prolonged periods of centrifugation (>24 h), and the use of salt-gradients that are dependent on operator proficiency with inconsistent reproducibility (Havel et al., 1955; Chapman et al., 1981). The FPLC-based methods for LDL enrichment used in this study provide an alternative method to ultracentrifugation-based LDL enrichment that presents none of the drawbacks seen with ultracentrifugation (Tauro et al., 2012). Altogether the LDL enrichment using FPLC to make NPs is a novel application of this method towards the synthesis of NPs and increases the translation potential of this technology.

*In vivo*, the safety profile of aposomes suggests very low toxicity as well as low immunogenicity. We compared the effect of aposomes on RBC rheological properties as a marker of RBC toxicity (Sosa et al., 2014) with liposomes using two complementary methods (ektacytometry and the AMVN device) and found no significant difference between the two types of NPs. Our findings suggest that both aposomes and liposomes have a similar safety profile concerning their effect on RBC mechanical properties *in vitro*. *In vivo*, analysis of aposomes’ effect on mice shows no alterations in metabolic profile, most notably in cholesterol serum levels. Furthermore, tissue architecture presented no notable signs of alteration, even when the aposome group was compared to untreated mice. In addition, aposome particles do not induce an immune response even when, either specific or non-specific, even after repeated exposures to high doses of aposomes in mice. These results underscore the safety profile of biomimetic lipid nanoparticles.

The targeting profile of our aposome platform shows a remarkable up to ∼20-fold increase in targeting over atherosclerotic plaques compared to liposomes. This increase is more than the two-fold increase seen with other biomimetic nanoparticles in previous studies (Martinez et al., 2018), which speaks to the distinct advantage that using LDL may provide over other transmembrane proteins. The different synthesis methods used in this study also shed light on the need to use different synthesis methods to create biomimetic nanoparticles. For example, while TLE has been by our lab functionalize membrane proteins onto lipid nanoparticles (Molinaro et al., 2016), microfluidic methods might be best suited to functionalize larger proteins onto the surface.

While there have been other nanoparticles developed to include LDL-derived peptides for targeting, the safety profile of the aposomes may provide an advantage in delivering drugs where safety limits the applications (such as rapamycin). The release kinetics seen for rapamycin in aposomes allows for prolonged release of this drug over several hours which could be helpful in maintaining the concentration within the therapeutic window for longer periods of time. In addition, the increased accumulation seen in plaques reduces the amount typically needed to reach a therapeutic effect which further reduces the toxicity of this drug increasing the possible application of otherwise toxic payloads. Future work should aim to expand the possible payloads (nucleic acids and proteins) and test the efficacy of this treatment with drug-loaded aposomes in a small animal models in larger numbers to validate this platform and reduce some of the variability seen in our study.

The methods described in this manuscript provide a novel method to purify and synthesize lipid nanoparticles with native apolipoproteins. Furthermore, the aposome’s safety and targeting capacities position this platform as a promising drug delivery method that could be used to develop new treatments for cardiovascular disease, especially for those therapeutic agents that are limited in their application by their toxicity.

## Data Availability

The raw data supporting the conclusions of this article will be made available by the authors, without reservation, upon request.
